# Random walk of passive tracers among randomly moving obstacles

**DOI:** 10.1186/s12976-016-0038-1

**Published:** 2016-04-14

**Authors:** Matteo Gori, Irene Donato, Elena Floriani, Ilaria Nardecchia, Marco Pettini

**Affiliations:** Aix-Marseille Université, Marseille, France; CNRS Centre de Physique Théorique UMR7332, Marseille, 13288 France; Centre d’Immunologie de Marseille-Luminy, Aix Marseille Université UM2, Inserm, U1104, CNRS UMR7280, Marseille, 13288 France

**Keywords:** Probability theory, Diffusion of biomolecules, Stochastic models in biological physics

## Abstract

**Background:**

This study is mainly motivated by the need of understanding how the diffusion behavior of a biomolecule (or even of a larger object) is affected by other moving macromolecules, organelles, and so on, inside a living cell, whence the possibility of understanding whether or not a randomly walking biomolecule is also subject to a long-range force field driving it to its target.

**Method:**

By means of the Continuous Time Random Walk (CTRW) technique the topic of random walk in random environment is here considered in the case of a passively diffusing particle among randomly moving and interacting obstacles.

**Results:**

The relevant physical quantity which is worked out is the diffusion coefficient of the passive tracer which is computed as a function of the average inter-obstacles distance.

**Conclusions:**

The results reported here suggest that if a biomolecule, let us call it a test molecule, moves towards its target in the presence of other independently interacting molecules, its motion can be considerably slowed down.

## Background

The topic of random walk in random environment (RWRE) has been the object of extensive studies during the last four decades and is of great interest to mathematics, physics and several applications. There is a huge literature on numerical, theoretical, and rigorous analytical results. The subject has been pioneered both through applications, as is the case of the models introduced to describe DNA replication [1], or through more abstract models in the field of probability theory [2]. One can find in Ref. [3] the definition of the mathematical framework of RWRE and since then a vast body of results has been built for both static and dynamic random environments, to mention just a few of them see [4–7] and the references therein quoted.

An example of biophysical application of RWRE is related with single-particle tracking experiments allowing to measure the diffusion coefficient of an individual particle (protein or lipid) on the cell surface; the knowledge of single-trajectory diffusion coefficient is useful as a measure of the heterogeneity of the cell membrane and requires to model hindered diffusion conditions [8].

To give another example among a huge number of processes in living matter, during B lymphocyte development, immunoglobulin heavy-chain variable, diversity, and joining segments assemble to generate a diverse antigen receptor repertoire. Spatial confinement related with diffusion hindrance from the surrounding network of proteins and chromatin fibres is the dominant parameter that determines the frequency of encounters of the above mentioned segments. When these particles encounter obstacles present at high concentration, the particles motions become subdiffusive [9] as described by the continuous time random walk (CTRW) model [8, 10].

In a biophysical context this kind of problems is referred to as “macromolecular crowding” which, among other issues, encompasses the effects of excluded volume on molecular diffusion and biochemical reaction rates within living cells. This problem has been largely studied both experimentally and numerically over the years (see respectively [11, 12] and references therein).

In this paper, we consider a very simplified model in order to obtain analytical results on the diffusion coefficient of passive tracers evolving among interacting and randomly moving particles. The prospective reason for studying this problem stems from the need of estimating how the encounter time of a given macromolecule (passive tracer) with its cognate partner, say a transcription factor diffusing towards is target on the DNA, is affected by the surrounding particles intervening in other biochemical reactions.

The complexity of real crowded systems appears at the moment very difficult to be managed by analytical calculations, for these reasons we have made important simplifications with respect to the realistic case. In particular, we have limited our analysis to a low concentration limit for the obstacles, assuming that the average distances among the particles (both tracers and obstacles) is much larger than their characteristic dimensions. Although this assumption is unrealistic in vivo, the present work can be considered as a first step in a feasibility study for an experiment oriented to infer whether intermolecular electrodynamic long range forces are at work in living matter using dilute solutions of biomolecules in vitro. This is in the same line as some recent works ([13–16]).

## Methods: continuous time random walk formalism

One of the many ways of modelling diffusive behavior is by Continuous Time Random Walk (CTRW) [17, 18]. This framework is mainly used to extend the description of Brownian motion to anomalous transport, in order to deal with subdiffusive or superdiffusive behavior in connection with Lévy processes, but it can of course be used to describe the simpler and more frequent case of normal diffusion. In this paper, we focus on cases where diffusion of tracers and interacting molecules is indeed Gaussian, so that a diffusion coefficient can be defined.

Consider a population of independent particles *A*, and suppose that their motion can be modeled as a sequence of motional events that take place in euclidean three dimensional space and in continuous time. In the literature (see for example [17]) calculations are often carried out in one dimension, however, the extension to two and three dimensions is trivial. In the CTRW framework the random walk is specified by *ψ*(**r**,*t*), the probability density of making a displacement **r** in time *t* in a single motional event. The normalization condition on *ψ*(**r**,*t*) is

(1) $$ \int_{0}^{+\infty} dt \int d^{3}\textbf{r} \;\psi(\textbf{r}, t) = 1  $$

In many applications of CTRW *ψ*(**r**,*t*) is decoupled so that there is no correlation between the displacement **r** and the time interval *t*:

(2) $$  \psi(\textbf{r}, t) = \Lambda(\textbf{r})\,\psi(t)  $$

Here we rather consider the formulation where space and time are coupled, thus expressing the fact that the particles move with a given velocity during single motional events; this amounts to introducing a conditional probability *p*(**r**|*t*), i.e., the probability that a given displacement **r** takes place in a time *t*

(3) $$  \psi(\textbf{r}, t) = \Lambda(\textbf{r})\; p(\textbf{r}| t) = \Lambda(\textbf{r})\; \delta\left(t - \frac{\left|\textbf{r}\right|}{\left|\textbf{v}(\textbf{r})\right|} \right)  $$

Normalization requires that

(4) $$ \int d^{3}\textbf{r} \;\Lambda(\textbf{r}) = 1  $$

We take the velocity to be constant in magnitude

(5) $$ \psi(\textbf{r}, t) = \Lambda(\textbf{r})\; \delta\left(t - \frac{\left|\textbf{r}\right|}{v_{0}}\right)  $$

Furthermore, we consider isotropic systems, which implies that the distribution *Λ*(**r**) is a function of *r*=|**r**| only. We write it in the form

(6) $$ \Lambda(\textbf{r}) = \frac{\lambda(r)}{4\pi r^{2}}  $$

with the normalization condition

(7) $$ \int_{0}^{+\infty} dr\;\lambda(r) = 1  $$

which allows to rewrite *ψ*(**r**,*t*) as

(8) $$  \psi(\textbf{r},t) = \frac{\lambda(r)}{4\pi\,r^{2}}\,\delta\left(t - \frac{\left|\textbf{r}\right|}{ v_{0}}\right) = \frac{\phi(t)}{4\pi\,(v_{0} t)^{2}}\,\delta\left(\left|\textbf{r}\right| - v_{0} t\right)  $$

where *ϕ*(*t*) is the free-flight or waiting time distribution representing the probability density function for a random walker to keep the same direction of its velocity during a time *t*. *ϕ*(*t*) is the fundamental quantity for the description of our isotropic system. It satisfies the relations

(9) $$  \phi(t) = v_{0} \, \lambda(v_{0} t)\;,\qquad\int_{0}^{+\infty} dt \,\,\phi(t) = 1  $$

Starting from these quantities, one can compute the Fourier-Laplace transform of the probability density *P*(**r**,*t*) for a particle to be at the position **r** at time *t*, and consequently calculate the diffusion properties. This is done in the Appendix, where we generalise the analysis carried out in [17] for the one-dimensional case to three dimensions, considering two slightly different versions of the CTRW:

- The Velocity Model, in which each particle *A* moves with constant velocity *v*_0_ between two turning points; at a turning point, a new direction and a new length of flight are taken according to the probability density *Λ*(**r**).
- The Jump Model, in which each particle waits at a particular location before instantaneously moving to the next one, the displacement being chosen according to the probability density *Λ*(**r**), the waiting time for a jump to take place being |**r**|/*v*_0_.

The expression of *P*(**r**,*t*) is formally different for these two versions of the CTRW, but from their definition it appears that the two models are equivalent in the long time limit.

As a general remark on other possible applications of our work, this CTRW description where space and time are coupled (see Eq. (3)) allows us to model situations not only of Gaussian diffusion but also of enhanced diffusion (where 〈*r*^2^(*t*)〉≃*t*^*α*^ with *α*>1) [18], because it can describe cases where the particles keep the same velocity for very long times (if the free-flight distribution *ϕ*(*t*) decays slowly, typically as an inverse power law).

We get normal diffusion as soon as *ϕ*(*t*) has a finite second moment. In this case, the long time behavior of the mean square displacement, and hence of the diffusion coefficient, is, both for the Velocity and Jump models (see the Appendix)

(10) $$  \left\langle r^{2}(t)\right\rangle = \int d^{3}\textbf{r}\; |\textbf{r}|^{2} P(\textbf{r}, t) \simeq \frac{{v_{0}^{2}}\langle t^{2}\rangle_{\phi}}{\langle t\rangle_{\phi}}\;t  $$

where 〈*t*^*n*^〉_*ϕ*_ is the *n*-th moment of the distribution *ϕ*(*t*) defined by Eqs. (8) and (9):

(11) $$ \langle t^{n}\rangle = \int_{0}^{+\infty} dt\,t^{n}\phi(t)  $$

The diffusion coefficient is then given by

(12) $$  D = {\lim}_{\textit{t}\rightarrow\infty} \frac{\langle r^{2}(t)\rangle}{6t} =\frac{{v_{0}^{2}}\langle t^{2}\rangle_{\phi}}{6\langle t\rangle_{\phi}}  $$

Let us notice that the same CTRW formalism can also describe subdiffusion (where 〈*r*^2^(*t*)〉≃*t*^*α*^ with *α*<1) [18]. This can be obtained by considering a version of the Jump Model where space and time are decoupled, as in Eq. (2): particles remain at a particular location for times distributed according to *ψ*(*t*) and make instantaneous jumps on distances distributed according to *Λ*(**r**). Subdiffusion is obtained as soon as *Λ*(**r**) has finite second moment while the first moment of the waiting time distribution *ψ*(*t*) diverges.

## Results and discussion

### Diffusion of independent tracers in the presence of interacting obstacles

If we adopt the CTRW description of diffusion presented in the preceding section, then the main quantity to consider is *ϕ*_*A*_(*t*), the probability density function that a random walker *A* keeps the same direction of velocity during a time *t*.

We will refer to “unperturbed” diffusion if *A* is the only species present in a solution, and we denote the free-flight time distribution of the unperturbed case by $\phi _{0_{A}}(t)$. The discussion in the preceding section gives

(13) $$  D_{0_{A}}= \frac{v_{0_{A}}^{2}\langle t^{2}\rangle_{\phi_{0_{A}}}}{6\langle t\rangle_{\phi_{0_{A}}}}\;  $$

which is also independently given by Einstein’s relation

(14) $$  D_{0_{A}} = \frac{kT}{\gamma_{A}}  $$

where *k* is the Boltzmann constant, *T* is the temperature and *γ*_*A*_ is the friction coefficient for *A*-particles given by Stokes’ Law:

(15) $$  \gamma_{A}=6\pi\,R_{A}\,\eta  $$

where *R*_*A*_ is the hydrodynamic radius of the diffusing particles and *η* is the viscosity of the medium where the particles diffuse.

Moreover, we can estimate the typical particle velocity using equipartition of energy

(16) $$  v_{0_{A}}^{2} = \frac{3kT}{m_{A}}  $$

where *m*_*A*_ is the mass of a particle *A*.

So, if we interpret $\phi _{0_{A}}(t)$ as the free-flight time distribution between Brownian collisions of the particles *A* on the molecules of the medium, then Eqs. (13), (14), (16) imply that the two first moments of $\phi _{0_{A}}$ must satisfy the relation

(17) $$  \frac{\langle t^{2}\rangle_{\phi_{0_{A}}}}{2\langle t\rangle_{\phi_{0_{A}}}} = \frac{m_{A}}{\gamma_{A}}  $$

As stated in the introduction, the physical situation we are interested in is the one where another population of particles, say *B*-particles, is also present in the solution. Particles *B* are supposed to diffuse and mutually interact, but there is no interaction at a distance between them and the particles *A*. It is reasonable to suppose that the diffusive and dynamic properties of these moving obstacles *B* induce changes in the diffusive properties of the *A*-particles which can be thus seen as passive tracers.

We want to model how the *B*-particles affect the diffusion properties of the *A*-particles by resorting to a suitable modification of the CTRW probability distribution *ϕ*_*A*_(*t*). The amount of the modification will of course depend on the concentration *C*_*B*_ (or equivalently on the average distance $d=C_{B}^{-1/3}$) of obstacles. Our goal is to estimate with simple arguments the dependence on the average distance *d* between any pair of obstacles of the ratio $D_{A}/D_{0_{A}}\phantom {\dot {i}\!}$ between perturbed and unperturbed diffusion coefficients.

We always assume that

(18) $$ C_{A} \ll C_{B}  $$

so that the *A*-particles can be regarded as tracers: any *A*-particle does not influence the dynamics of the obstacles and of the other tracers.

### Modification of the microscopic free-flight time distribution

If the concentration *C*_*B*_ of the obstacles *B* is low enough, in the sense that their average distance *d* is such that

(19) $$ d\gg \sqrt{\int_{0}^{+\infty} dr \; r^{2} \lambda_{0}(r)}  $$

we can consider that the diffusion of *A*-particles is not perturbed by the presence of the obstacles *B*; thus for the waiting time distribution we will have $\phantom {\dot {i}\!}\phi _{A}(t)\simeq \phi _{0_{A}}(t)$, and, consequently, $\phantom {\dot {i}\!}D_{A}\simeq D_{0_{A}}$.

As the concentration of *B*-particles grows, the diffusion of *A*-particles is affected accordingly, and this is described by a modification of *ϕ*_*A*_(*t*). It is reasonable to suppose that *ϕ*_*A*_(*t*) will be close to $\phi _{0_{A}}(t)$ at sufficiently short times, i.e., for displacements small enough that a tracer *A* does not “see” any obstacle *B*, and that *ϕ*_*A*_(*t*) will be reduced with respect to the unperturbed $\phi _{0_{A}}(t)$ at long times, because long free displacements are likely to be interrupted by the presence of obstacles.

Following this idea, we model the waiting time distribution as follows: we call *T*_*d*_ the characteristic time of flight at which a tracer *A* begins to “see” the obstacles *B*, where *T*_*d*_ depends of course on the typical distance $d\simeq C_{B}^{-1/3}$ between the *B*-particles. We then make the simplest assumption that *ϕ*_*A*_(*t*) coincides (except for a normalisation factor) with $\phi _{0_{A}}(t)$ for times smaller than *T*_*d*_ and is zero for times larger than *T*_*d*_.

We take the unperturbed distribution $\phi _{0_{A}}(t)$ to be exponentially decreasing

(20) $$  \phi_{0_{A}}(t) = \frac{1}{\tau_{A}}\,e^{-t/\tau_{A}}  $$

where, using Eq. (17):

(21) $$  \tau_{A}=\frac{m_{A}}{\gamma_{A}}  $$

So, we write the modified probability density *ϕ*_*A*_(*t*) for passive tracers (*A*-particles) in presence of interacting moving obstacles (*B*-particles) as:

(22) $$  \phi_{A}(t) = \frac{e^{-t/\tau_{A}}}{\tau_{A}(1-e^{-T_{d}/\tau_{A}})}\;\;\;\;\;\text{if}\;\; t<T_{d}\;,\qquad \phi_{A}(t)=0\quad\text{if}\;\; t \ge T_{d}  $$

If we compute the diffusion coefficient using Eq. (12) and expressions (22), (20) we get

(23) $$  \frac{D_{A}}{D_{0_{A}}} = \frac{\langle t^{2}\rangle_{\phi_{A}}}{\langle t\rangle_{\phi_{A}}}\cdot\frac{\langle t\rangle_{\phi_{0_{A}}}}{\langle t^{2}\rangle_{\phi_{0_{A}}}} = 1 - \frac{x^{2}}{2\,(e^{x} -1 -x)}\quad\text{where}\;\;x=x(d)=\frac{T_{d}}{\tau_{A}}  $$

which is a function of the ratio between the transition time *T*_*d*_ and the characteristic timescale *τ*_*A*_ of the non perturbed waiting time distribution. The issue is now to establish the dependence of the transition time *T*_*d*_ (and consequently, of the parameter *x*) on the average distance *d* between obstacles.

The fact that the obstacles move under the influence of deterministic nonlinear interparticle potentials implies a chaotic dynamics which a-priori could be very different from a stochastic dynamics, this notwithstanding such a chaotic dynamics entails a Brownian-like diffusion as was found by numerical simulations in Ref. [14]. Hence we assume that the *B*-molecules (obstacles) diffuse with Brownian motion: we can apply to them the CTRW description with velocity $v_{0_{B}}$ and waiting time distribution *ϕ*_*B*_(*t*), corresponding to a situation where they do not interact. We can then approximately take into account their mutual interaction by giving them a systematic drift velocity that is due to deterministic forces acting between them. This drift velocity depends on their mutual distance *d*, and we will call it *V*_*d*_. If we suppose that the dynamics of the *B*-molecules is over-damped, a crude estimation of *V*_*d*_ is given by *V*_*d*_≃*F*(*d*)/*γ*_*B*_, where *γ*_*B*_=6*πR*_*B*_*η* is the friction coefficient of the *B*-molecules and *F*(*d*) the norm of the deterministic force between two molecules of type *B* at a distance $d=C_{B}^{-1/3}$.

The transition time *T*_*d*_ can be roughly estimated by considering that, if the diffusive displacement of a tracer *A* is interrupted by the presence of the *B*-molecules, this is due to a molecule *B* which is moving in the direction of the tracer *A*, so that

(24) $$  T_{d} \simeq \frac{d}{v_{0_{A}} + v_{0_{B}} + V_{d}} \simeq \frac{d}{v_{0_{A}} + v_{0_{B}} + F(d)/\gamma_{B}}  $$

For the parameter *x* appearing in (23) this gives

(25) $$  x=x(d)=\frac{T_{d}}{\tau_{A}}=\frac{d}{\frac{m_{A}}{\gamma_{A}} \left[ \sqrt{\frac{3kT}{m_{A}}} + \sqrt{\frac{3kT}{m_{B}}} + \frac{F(d)}{\gamma_{B}}\right]}  $$

where we have used Eqs. (16), (17) and (20).

Now, some remarks are in order. The most tricky point in the procedure mentioned above to compute $D_{A}/D_{0_{A}}\phantom {\dot {i}\!}$ of the tracers consists in the choice of the functional form of *T*_*d*_=*T*_*d*_[*U*(*r*)](*d*), where *U*(*r*) is the potential energy among interacting obstacles which depends only on the distance *r* between them, i.e.

(26) $$ F(r)=\left|\frac{\mathrm{d} U}{\mathrm{d} r}(r)\right|  $$

Equation (24) is a rough estimate of this characteristic time because it excludes, for instance, effects due to the dimensionality of physical space where diffusion takes place (1D, 2D, etc.), the sign of interaction energy among obstacles, spatial correlation among obstacles and the possibility of multiple collisions among the molecules. The last point entails the exclusion - from the range of validity of our model - of all the cases where *d*≲ min{*R*_*A*_,*R*_*B*_} (as in the case of densely crowded systems). For this reason we do not take into account the sizes of both tracers and obstacles at a distance *d* from the colliding particle.

Moreover, this model is meaningful if the transition time *T*_*d*_ is of the same order of magnitude than the characteristic timescale *τ*_*A*_ of *ϕ*_*A*_(*t*). Such a condition is equivalent to requiring that the viscosity *η* of the medium and the interparticle distance *d* are sufficiently small and, possibly, the interaction strength among the obstacles is sufficiently large. To the contrary, if the parameters of the system are such that the typical time *T*_*d*_ at which the tracers “see” the obstacles is many orders of magnitude larger than the typical time *τ*_*A*_ between Brownian collisions, the free-flight time distribution $\phi _{0_{A}}(t)\phantom {\dot {i}\!}$ will not be modified by the presence of the obstacles, and Eq. (23) will always give $D_{A}\simeq D_{0_{A}}\phantom {\dot {i}\!}$, as *x*(*d*)≫1 for all the accessible values of *d*. More precisely, if we look at Eq. (25) for the ratio between *T*_*d*_ and *τ*, it is reasonable to think that the presence of *B*-particles modifies the microscopic free-flight time distribution between Brownian collisions if the product (*γ*_*A*_*d*) is not much larger than $\sqrt {m_{A} k T}$. Unfortunately this is not true in many applications. Consider, for instance, the case of two molecular species diffusing in water (*η*=5.1×10^8^ KDa *μm*^−1^*μs*^−1^) at room temperature *T*=300 K, where the *A*-particles are non interacting small molecules (say a small peptide complex), and the *B*-particles represent mutually interacting biomolecules with *m*_*B*_≃20 KDa and *R*_*B*_≃2×10^−3^*μ*m, so that *R*_*A*_≃0.5 *R*_*B*_ and *m*_*A*_≃0.025 *m*_*B*_≃0.5 KDa. Using Eqs. (21) and the previous choice of physical parameters for *A*-particles, we obtain that *τ*_*A*_≃5×10^−8^*μ*s. Suppose that the *B*-particles are characterized by a net electric charge *Z*_*B*_≃10, that their mutual average distance is *d*=0.05 *μ*m ≃50 *R*_*B*_, and that they interact through a non screened electrostatic potential. This models the case of an ideal watery solution of *A*- and *B*-type particles with no Debye screening, and with *ε*_*rel*_≃80 (the value of the static dielectric constant of water). Using Eq. (16) we see that the contribution due to thermal noise of *A*-type molecules is larger than that of the *B*-type molecules, in fact $v_{0_{A}} \simeq 1.2\times 10^{2}\mu \mathrm {m}\mu \mathrm {s}^{-1} \simeq 6\, v_{0_{B}}$; moreover, the interaction term is negligible with respect to the velocities, as

(27) $$ \frac{F(d)}{\gamma_{B}}=\frac{{Z_{B}^{2}} q^{2}}{\varepsilon_{rel} d^{2}}\frac{1}{\gamma_{B}}\simeq 7 \times10^{-3}\mu m \mu s^{-1} \simeq 3\times 10^{-3} v_{0_{B}}  $$

where *q* is the elementary charge expressed in Gaussian units. Using (24), the transition time is *T*_*d*_≃3·10^−4^*μ*s, whence we get *x*(*d*)≃6·10^3^.

### Modification of the rescaled free-flight time distribution

In order to describe physical systems for which *T*_*d*_≫*τ* for all the accessible values of the intermolecular distance *d*, as the one described by the preceding example, we have to modify the CTRW model.

Let us still model the unperturbed diffusion of tracers as a sequence of linear motional events described in the CTRW formalism by a rescaled function $\tilde {\psi }_{0_{A}}(\textbf {r},t)$, given by

(28) $$  \tilde{\psi}_{0_{A}} (\textbf{r},t) = \frac{1}{4\pi\,(\tilde{v}_{0_{A}} t)^{2}}\,\tilde{\phi}_{0_{A}}(t)\,\delta(\left|\textbf{r}\right| - \tilde{v}_{0_{A}} t) = \frac{1}{4\pi\,(\tilde{v}_{0_{A}} t)^{2}}\,\frac{e^{-t/\tilde{\tau}_{A}}}{\tilde{\tau}_{A}}\,\delta(\left|\textbf{r}\right| - \tilde{v}_{0_{A}} t)  $$

where $\tilde {v}_{0_{A}}=\alpha _{A} v_{0_{A}}$ is a rescaled velocity and $\tilde {\tau }_{A}=\beta _{A}\tau _{A}$ is a rescaled characteristic timescale for diffusive motional events. The parameters $v_{0_{A}}$ and *τ*_*A*_ are the same as in the previous section. If there are no interactions among obstacles (*B*-particles), a relation equivalent to Eq. (28) can be written for each *B* particle

(29) $$  \tilde{\psi}_{0_{B}} (\textbf{r},t) = \frac{1}{4\pi\,(\tilde{v}_{0_{B}} t)^{2}}\,\tilde{\phi}_{0_{B}}(t)\,\delta(\left|\textbf{r}\right| - \tilde{v}_{0_{B}} t) = \frac{1}{4\pi\,(\tilde{v}_{0_{B}} t)^{2}}\,\frac{e^{-t/\tilde{\tau}_{B}}}{\tilde{\tau}_{B}}\,\delta(\left|\textbf{r}\right| - \tilde{v}_{B_{0}} t)  $$

where, analogously to the previous case, $\tilde {v}_{0_{B}}=\alpha _{B} v_{0_{B}}$ and $\tilde {\tau }_{B}=\beta _{B}\tau _{B}$.

Of course this does not model the microscopic level, in the sense that the single motional events - whose probability is specified by $\tilde {\psi }_{A} (\textbf {r},t)$ - are no longer the microscopic displacements between successive Brownian collisions. Rather, we focus on the motion on longer timescales $\tilde {\tau }_{A}$ (*β*_*A*_>1) and model the diffusion of tracers as a sequence of displacements on typical distances $\tilde {v}_{A_{0}}\tilde {\tau }_{A}$.

The conditions on the rescaling parameters (*α*_*A*_,*β*_*A*_,*α*_*B*_,*β*_*B*_) are then

- the typical motional event for tracers (*A*-particles) takes place between two consecutive encounters with an obstacle (*B*-particles); this means that the spatial scale of a typical motional event for tracers described by $\tilde {\psi }_{0_{A}}(\textbf {r},t)$ is *d*, the average distance between any two obstacles. This condition guarantees that *τ*_*A*_, and consequently $\tilde {\psi }_{0_{A}}(\textbf {r},t)$, is modified in the presence of obstacles:
  (30) $$  \left(\tilde{v}_{0_{A}}+\tilde{v}_{0_{B}}\right)\tilde{\tau}_{A} = \left(\alpha_{A} v_{0_{A}}+\alpha_{B} v_{0_{B}}\right)\beta_{A} \tau_{A}= d  $$
- for *B*-particles we can also write a condition analogous to Eq. (30) under the assumption that the motional events for obstacles are determined by encounters among them in absence of mutual interactions. This is justified by the assumption that the concentration of tracers is negligible compared with the concentration of obstacles. In this framework it is reasonable to assume:
  (31) $$  2\tilde{v}_{0_{B}}\tilde{\tau}_{B}=2\alpha_{B} \beta_{B} \left(v_{0_{B}}\tau_{B}\right)=d  $$
- the dynamics of tracers is now dominated by the encounters with obstacles, that means
  (32) $$  \frac{\tilde{v}_{0_{A}}^{2}\tilde{\tau_{A}}}{3}=D_{exVol_{A}}(d)  $$
  where $D_{exVol_{A}}(d)$ is the diffusion coefficient of tracers taking into account the excluded volume effects due to the presence of the obstacles. As we are investigating the case *d*≫*R*_*A*_+*R*_*B*_, we can neglect the excluded volume effects and substitute $D_{0_{A}}=D_{exVol_{A}}(\infty)$, yielding:
  (33) $$  \frac{\tilde{v}_{0_{A}}^{2}\tilde{\tau_{A}}}{3} = \frac{{\alpha_{A}^{2}} \beta_{A} \left(v_{0_{A}}^{2}\tau_{A}\right)}{3} = \frac{v_{0_{A}}^{2}\tau_{A}}{3}=D_{0_{A}} \;\;\;\; \Rightarrow \;\;\;\; {\alpha_{A}^{2}}\beta_{A} =1 \  $$
- the considerations in the previous item can be extended to obstacles (*B*-particles) if no interactions act among them, so that:
  (34) $$  \frac{\tilde{v}_{0_{B}}^{2}\tilde{\tau_{B}}}{3} = \frac{{\alpha^{2}_{B}}\beta_{B}\left(v_{0_{B}}^{2}\tau_{B}\right)}{3} = \frac{v_{0_{B}}^{2}\tau_{B}}{3}=D_{0_{B}} \;\;\;\; \Rightarrow \;\;\;\; {\alpha_{B}^{2}}\beta_{B} =1 $$

Notice that the rescaled velocity and time now implicitly depend on the parameter *d*.

Solving the system formed by Eqs. (30), (31), (33) and (34), we obtain:

(35) $$ \alpha_{B}=\frac{2\, v_{0_{B}} \tau_{B}}{d} \qquad \beta_{B}=\frac{1}{{\alpha_{B}^{2}}}  $$

while for the rescaled parameters for *A*-particles:

(36) $$ \alpha_{A}=\frac{v_{0_{A}}\tau_{A}}{2d}\left(1\pm\sqrt{1+8\,\frac{v_{0_{B}}^{2}\tau_{B}}{v_{0_{A}}^{2}\tau_{A}}}\right) \qquad \beta_{A}=\frac{1}{{\alpha_{A}^{2}}}  $$

where, as *α*_*A*_>0, the physical solution we choose is the one with the “ + ” sign.

Using Eqs. (16) and (21) we can rewrite this as:

(37) $$ \alpha_{B}=\frac{2\,\sqrt{3kTm_{B}}}{\gamma_{B} d} \qquad \beta_{B}=\frac{1}{{\alpha_{B}^{2}}}  $$

and

(38) $$  \alpha_{A}=\frac{\sqrt{3kTm_{A}}}{2\gamma_{A} d}\left(1 +\sqrt{1+8\,\frac{\gamma_{A}}{\gamma_{B}}}\right) \qquad \beta_{A}=\frac{1}{{\alpha_{A}^{2}}}  $$

We suppose that, in the presence of mutually interacting biomolecules of *B*-type, the function $\tilde {\phi }_{0_{A}}(t)$ is modified as follows

(39) $$  \tilde{\phi}_{A}(t) = q_{1}\,e^{-t/\tilde{\tau}_{A}} \quad\text{if}\;\; t<\tilde{T}_{d}\;,\qquad \tilde{\phi}_{A}(t) = q_{2}\,e^{-t/\tilde{T}_{d}} \quad\text{if}\;\; t\ge\tilde{T}_{d}  $$

where *q*_1_,*q*_2_ are such that $\tilde {\phi }_{A}(t)$ is normalized and continuous at $t=\tilde {T}_{d}$. $\tilde {T}_{d}$ is again the characteristic time at which the motional events described by $\tilde {\psi }_{0_{A}}(\textbf {r},t)$ are perturbed by the presence of the obstacles. Equation (39) expresses the fact that, on spatial scales larger than the average intermolecular distance *d* between any pair of obstacles, the timescale of diffusion changes from $\tilde {\tau }_{A}$ to $\tilde {T}_{d}$, which is the characteristic time that takes to cover a distance *d* for a tracer in presence of interacting obstacles. Two physically equivalent conditions for defining $\tilde {T}_{d}$ are

(40) $$  \tilde{T}_{d} \simeq \frac{d}{\tilde{v}_{0_{A}}+\tilde{v}_{0_{B}}+ V_{d}}  $$

Here *V*_*d*_ is the drift velocity of the obstacles, that we can estimate in the same way as in Section “Modification of the microscopic free-flight time distribution”, that is, *V*_*d*_≃*F*(*d*)/*γ*_*B*_. For both conditions, it is evident that $\tilde {T}_{d}\le \tilde {\tau }_{A}$, where the equality holds when *V*_*d*_=0, that is, the *B*-particles do not interact.

After a straightforward calculation, we obtain the following dependence of the diffusion coefficient on the parameter $y(d)= \tilde {T}_{d}/\tilde {\tau }_{A}$:

(41) $$  \frac{D_{A}}{D_{0_{A}}} = \frac{1 - e^{-y} \left(1+y+\frac{y^{2}}{2}-\frac{5y^{3}}{2} \right)}{1 - e^{-y}\left(1+y-2y^{2}\right)}  $$

If we take the condition (40) for $\tilde {T}_{d}$ we get for the dependence of *y* on *d*

(42) $$  y(d) = \frac{\tilde{T}_{d}}{\tilde{\tau}_{A}} = \frac{1}{1 + \frac{V_{d}\,\tilde{\tau}_{A}}{d}} = \frac{1}{1 + \frac{F(d)}{d \gamma_{B}}\beta_{A}\frac{m_{A}}{\gamma_{A}}} = \frac{1}{1 + \frac{4 d \,F(d)\gamma_{A}}{3 k T\gamma_{B} \left(1 +\sqrt{1+8\,\frac{\gamma_{A}}{\gamma_{B}}}\right)^{2}}}  $$

where we have used Eq. (38) for *β*_*A*_.

### Slowing down of Brownian diffusion: the patterns of *D*/*D*_0_

In this section we report the patterns of the ratio $D_{A}/D_{0_{A}}\phantom {\dot {i}\!}$ obtained by means of the theoretical expressions (23), (25) and (41), (42). We denote by *D* and *D*_0_ the diffusion coefficients of the tracers (*A*-particles) in the presence and in the absence of obstacles (*B*-particles), respectively. We plot this ratio as a function of the average distance *d* between any two obstacles obtained for different kinds of interaction potentials between the *B*-particles: screened electrostatic potential, Coulombic potential, dipolar potential. These potentials have been chosen as they are representative of some relevant interaction in biology [19]. The choice of Coulombic and dipolar potentials is justified by the fact that these are long range interactions that can exert their action on a length scale much larger than the typical dimensions of biomolecules. In this framework other interactions, i.e. Van der Waals interactions, have a very short range and they exert their action on length scale comparable with biomolecules dimensions. Nevertheless the short range screened Coulombic potential has been investigated as its range distance depends on the free ions concentration in the diffusive medium, which is an accessible experimental parameter. In what follows the diffusion of tracers in presence of interacting obstacles is studied for some cases corresponding to the different frameworks discussed in Sections “Modification of the microscopic free-flight time distribution”, “Modification of the rescaled free-flight time distribution”.

### Case of modification of the microscopic free-flight time distribution

As discussed in Section “Modification of the microscopic free-flight time distribution”, this approach corresponds to the case where the characteristic timescale *τ*_*A*_ of Brownian collisions is of the same order of magnitude than *T*_*d*_ (the characteristic timescale at which the tracers *A* “see” the obstacles *B*). This corresponds to intermolecular distances *d* of the obstacles that are comparable to $\sqrt {\frac {m_{A} k T}{{\gamma _{A}^{2}}}}\,$. For the sake of simplicity we consider the case where the species *A* and *B* have the same size, *R*=*R*_*A*_=*R*_*B*_, and the same mass, *m*=*m*_*A*_=*m*_*B*_, which define a length and a mass scale for the system, respectively. Hence, for instance, the distance between two colliding particles can be rewritten as *d*=*Rl*, where *l* is an adimensional parameter, with the assumption that *d*≫*R*. Moreover, the temperature *T* of the system defines an energy scale allowing to express Eq. (25) in terms of dimensionless quantities, since the friction coefficient as well can be expressed in terms of an adimensional parameter *Γ*

(43) $$ \gamma=\Gamma(k T m)^{1/2}R^{-1}  $$

Let us consider a two-body interaction potential of the form

(44) $$  U(r)=\frac{\mathcal{C}}{r^{n}}  $$

where *r* is the interparticle distance, which can be written in adimensional units as

(45) $$  U(r=Rl)=\bar{U}(l)=(k T) \bar{\mathcal{C}} l^{-n}  $$

where $\bar {\mathcal {C}}=\mathcal {C} (k T R^{n})^{-1}$. With these conventions, Eq. (25) reads

(46) $$  x=x(d=Rl)=\frac{T_{d}}{\tau}=\frac{l\Gamma^{2}}{2\sqrt{3}\Gamma +\bar{\mathcal{C}} n l^{-(n+1)}}  $$

Let us consider the case of a Coulombic interaction

(47) $$ U_{Coul}(r)=\mathcal{C}_{Coul}\, r^{-1}  $$

among *B*-type particles. In order to study a somewhat realistic case we take for *m* and *R* values that are typical for macromolecules, i.e. *m*∼10 KDa≃1.6×10^−23^ Kg, *R*≃10^−9^ m and |*Z*|≃10, at room temperature *T*=300 K; in this case we have

(48) $$  \bar{\mathcal{C}}_{Coul}=\frac{Z^{2} q^{2}}{\varepsilon_{water} (kTR)}\simeq 0.7\times 10^{2}  $$

where *q* is the electric elementary charge and *ε*_*water*_≃80 is the relative electric permittivity of water.

**Fig. 1:**
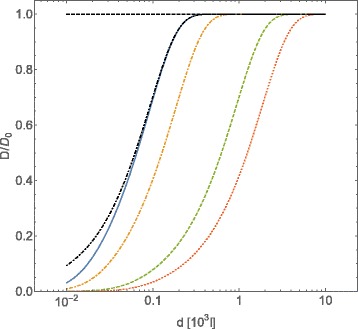
Normalized diffusion coefficient *D*/*D*_0_ for *A*-type particles, computed with Eqs. (23) and (25), plotted vs. the intermolecular average distance *d* of *B*-type particles (expressed in adimensional units *l*). The *B*-particles interact through a Coulombic potential $U=\bar {\mathcal {C}}_{Coul}l^{-1}$. The *A*- and *B*-type particles are assumed spherical, of equal radius *R*, and equal mass *m*. In adimensional units the interaction intensity is $\bar {\mathcal {C}}_{Coul}=U_{Coul}(R)/(k_{B}T)$, the friction coefficient *γ*=*Γ*(*k*_*B*_ *T* *m*)^1/2^ *R* ^−1^. The curves refer to a fixed value for the potential strength ($\bar {\mathcal {C}}_{Coul}=70$) and different values for *Γ*, that is: *Γ*=0.1 (*blue continuous line*), *Γ*=0.05 (*orange dot-dashed line*), *Γ*=0.01 (*green dashed line*), *Γ*=0.005 (*red dotted line*). The case of $\bar {\mathcal {C}}_{Coul}=0$ has been reported for *Γ*=0.01 (*black dot dashed line*)

In Fig. 1 we plot the tracer self-diffusion coefficent behavior as a function of average distances among diffusing obstacles interacting through a Coulombic potential, following Eqs. (23) and (46); the intensity of Coulombic potential has been fixed to $\bar {\mathcal {C}}_{Coul}=0.7\times 10^{2}$ while the value of the adimensionalized friction coefficient *Γ* has been changed. In this case it is necessary to choose *Γ*≃10^−2^ in order to obtain sizeable effects on the value of *D*/*D*_0_ at an average intermolecular distance of about *l*≃10^3^. Moreover, the value of *Γ* strongly affects the value of the intermolecular average distance among obstacles, which corresponds to a major deviation of the tracer self-diffusion coefficient from its Brownian value: the smaller the value of *Γ* is, the larger the distance among obstacles at which diffusion of tracers deviates from Brownian diffusion.

Assuming that the friction coefficient is given by Stokes’ law (15), the obtained *Γ* value corresponds to *η*≃1.5×10^−4^*η*_*water*_, where *η*_*water*_ is the viscosity of water at temperature *T*=300 K.

**Fig. 2:**
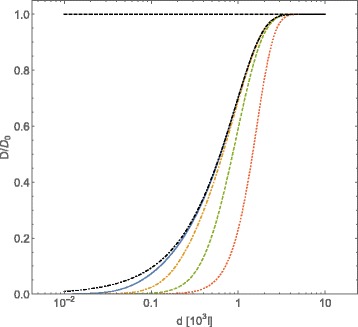
Normalized diffusion coefficient *D*/*D*_0_, computed with Eqs. (23) and (25), for *A*-type particles vs. intermolecular average distance *d* of *B*-type particles (expressed in adimensional units). The *B*-particles interact through a Coulombic potential $U_{Coul}=\bar {\mathcal {C}}l^{-1}$. Conventions on units are the same of Fig. 1. The curves refer to the fixed value *Γ*=0.01 of the friction coefficient, and to different values of the potential strength: $\,\bar {\mathcal {C}}_{Coul}=10^{2}$ (*continuous line*), $\,\bar {\mathcal {C}}_{Coul}=10^{3}$ (*dot-dashed line*), $\,\bar {\mathcal {C}}_{Coul}=10^{4}$ (*dashed line*), $\,\bar {\mathcal {C}}_{Coul}=10^{5}$ (*dotted line*). The case of $\bar {\mathcal {C}}_{Coul}=0$ has been reported (*black dot dashed line*)

In Fig. 2 we plot the tracers self-diffusion coefficient behavior as a function of the average distances among diffusing obstacles interacting through a Coulombic potential, for a fixed value of *Γ*=10^−2^ and different values of the strength of Coulombic interaction among obstacles. In this case we observe that, as we increase the strength of Coulombic potential, the profile of tracers self-diffusion coefficient as a function of the average distance among obstacles becomes sharper. Nevertheless, the intensity of the potential does not seem to affect the value of the average distance among obstacles at which the tracers self-diffusion coefficient deviates from its value in the absence of interactions.

We can conclude that the self-diffusion coefficient of tracers is mainly affected by the value of the friction coefficient. In the range of cases we have studied, the presence of interactions among obstacles affects only slightly the diffusion behavior of tracers, as it can be seen by comparing with the case $\bar {\mathcal {C}}_{Coul}=0$. This effect can be interpreted as a sort of “effective dynamical excluded volume” due to the presence of the obstacles; when the friction forces are weakened, the average speed both of the obstacles and the tracers increases and as a consequence the average free-flight time of tracers diminishes.

As mentioned above, the renormalized self-diffusion coefficient of tracers has been computed also in presence of obstacles interacting through a “dipole-dipole” potential

(49) $$ U_{Dip}(r)=\mathcal{C}_{Dip}\, r^{-3}  $$

and a screened Coulombic potential, of a form close to the Debye-Hückel potential (which usually models electrostatic interactions in electrolytic solutions), that is

(50) $$  U_{CoulScr}(r)=\frac{\mathcal{C}_{CoulScr}\exp\left[ -r/\lambda_{D} \right]}{r}  $$

where *λ*_*D*_ is the characteristic screening length scale, also called Debye length.

The potential in (50) can be rewritten in adimensional form:

(51) $$ U_{CoulScr}(r=Rl)=\bar{U}_{CoulScr}(l)=\frac{U_{Coul}(R)}{k T}\frac{\exp\left[-\frac{l-1}{\bar{\lambda}_{D}}\right]}{l}=\bar{\mathcal{C}}_{CoulScr}\frac{\exp\left[-\frac{l-1}{\bar{\lambda}_{D}}\right]}{l}  $$

where $\bar {\lambda }_{D}=\lambda _{D}/R$ is the adimensional screening length. As pointed out before, the method proposed in the present paper is meaningful provided that *d*≫*R*, therefore we take $\,\bar {\lambda }_{D} \geq 10$, since for shorter screening length scales we don’t expect any effect of the interactions among obstacles on the diffusion of tracers. For the screened Coulombic potential, Eq. (25) takes the form

(52) $$  x=x(d=Rl)=\frac{T_{d}}{\tau_{A}}=\frac{l\Gamma^{2}}{2\sqrt{3}\Gamma +\bar{\mathcal{C}}_{CoulScr} \left(\frac{1}{l^{2}}+\frac{1}{l\bar{\lambda}_{D}}\right)\exp\left(-\frac{l-1}{\bar{\lambda}_{D}}\right)}  $$

**Fig. 3:**
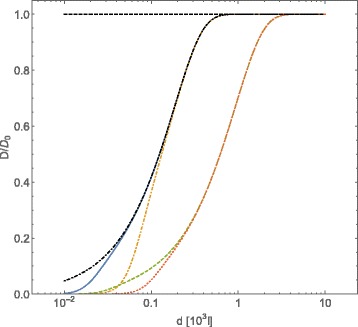
Normalized diffusion coefficient *D*/*D*_0_, computed with Eqs. (23) and (25), for *A*-type particles vs. intermolecular average distance *d* of *B*-type particles (expressed in adimensional units). The *B*-type particles interact through a dipolar potential $U(r)={\mathcal {C}}_{Dip} r^{-3}=\bar {\mathcal {C}}_{Dip}(r/R)^{-3}$. Conventions on adimensional units are the same of Fig. 1. The curves refer to different choices of the friction coefficient *Γ* and of the strength $\bar {\mathcal {C}}_{Dip}$ of the potential energy: $\Gamma =0.05 \,\,\text {and}\,\, \bar {\mathcal {C}}=10^{4}$ (*blue continuous line*), $\Gamma =0.05\,\,\text {and}\,\,\bar {\mathcal {C}}=10^{6}$ (*orange dot-dashed line*), $\Gamma =0.01\,\,\text {and}\,\,\bar {\mathcal {C}}=10^{4}$ (*green dashed line*), $\Gamma =0.01\,\,\text {and}\,\,\bar {\mathcal {C}}=10^{6}$ (*red dotted line*). The case of $\bar {\mathcal {C}}_{Dip}=0$ has been reported (*black dot dashed line*) for *Γ*=0.05

**Fig. 4:**
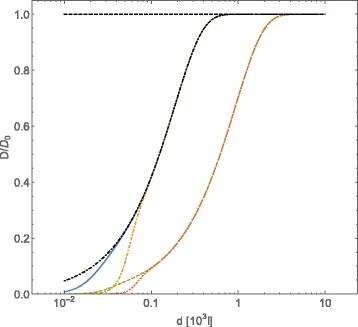
Normalized diffusion coefficient *D*/*D*_0_, computed with Eqs. (23) and (25), for *A*-type particles vs. intermolecular average distance *d* of *B*-type particles (expressed in adimensional units). The *B*-type particles interact through the Coulombic screened potential given in (50). Conventions on adimensional units are the same of Fig. 1. The curves refer to different choices of the value of the friction coefficient *Γ* and of the screening length *λ*_*D*_=10 which set the strength of the potential energy: $\,\Gamma =0.05 \,\,\text {and}\,\, \bar {\mathcal {C}}_{CoulScr}=10^{2}$ (*blue continuous line*), $\,\Gamma =0.05\,\,\text {and}\,\, \bar {\mathcal {C}}_{CoulScr}=10^{6}$ (*orange dot-dashed line*), $\,\Gamma =0.01\,\,\text {and}\,\, \bar {\mathcal {C}}=10^{2}$ (*green dashed line*), $\,\Gamma =0.01\,\,\text {and}\,\, \bar {\mathcal {C}}_{CoulScr}=10^{6}$ (*red dotted line*). The case of $\bar {\mathcal {C}}_{CoulSCr}=0$ has been reported (*black dot dashed line*) for *Γ*=0.05

In Figs. 3 and 4 we show the behavior of tracers self-diffusion coefficient as a function of the concentration of interacting obstacles, in the case of “dipolar” and Coulomb screened interactions among obstacles, respectively. Different values for *Γ*, $\bar {\mathcal {C}}_{Dip}$ and $\bar {\mathcal {C}}_{CoulScr}$ have been chosen. In both cases we observe that the dependence of the tracers self-diffusion coefficient on the concentration of obstacles is much more affected by the value of *Γ* than by the strength of the interaction potentials among obstacles $\bar {\mathcal {C}}_{Dip}$ and $\bar {\mathcal {C}}_{CoulScr}$, at least in the explored range of parameters. This allows to conclude that also in this case the “effective dynamical excluded volume” mainly affects the tracers self-diffusion coefficient.

### Case of modification of the rescaled free-flight time distribution

As discussed in Section “Modification of the rescaled free-flight time distribution”, the proposed approach corresponds to the case where the characteristic timescale *τ* of Brownian collisions is much smaller than the transition time *T*_*d*_. This corresponds to intermolecular distances *d* of the obstacles that are much larger than $\sqrt {\frac {m_{A} k T}{{\gamma _{A}^{2}}}}\,$.

**Fig. 5:**
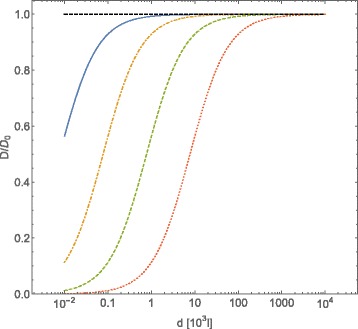
Normalized diffusion coefficient *D*/*D*_0_, computed with Eqs. (41) and (42), for *A*-type particles vs. intermolecular average distance *d* of *B*-type particles (expressed in adimensional units). The *B*-type particles interact through the Coulombic potential $U=\bar {\mathcal {C}}l^{-1}$. Conventions on adimentional units are the same of Fig. 1. The curves refer to different values of potential strength: $\bar {\mathcal {C}}_{Coul}=10^{2}$ (*continuous line*), $\,\bar {\mathcal {C}}_{Coul}=10^{3}$ (*dot-dashed line*), $\,\bar {\mathcal {C}}_{Coul}=10^{4}$ (*dashed line*), $\,\bar {\mathcal {C}}_{Coul}=10^{5}$ (*dotted line*). The flat dashed line correspond to the case $\bar {\mathcal {C}}_{Coul}=0$

**Fig. 6:**
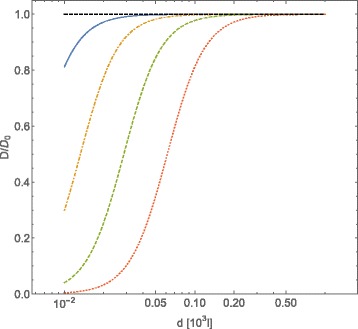
Normalized diffusion coefficient *D*/*D*_0_, computed with Eqs. (41) and (42), for *A*-type particles vs. intermolecular average distance *d* of *B*-type particles (expressed in adimensional units). The *B*-type particles interact through a dipolar potential $U=\bar {\mathcal {C}}l^{-3}$. Conventions on adimensional units are the same of Fig. 1. The curves refer to different values for potential strength: $\,\bar {\mathcal {C}}_{Dip}=10^{2}$ (*continuous line*), $\,\bar {\mathcal {C}}_{Dip}=10^{3}$ (*dot-dashed line*), $\,\bar {\mathcal {C}}_{Dip}=10^{4}$ (*dashed line*), $\,\bar {\mathcal {C}}_{Dip}=10^{5}$ (*dotted line*). The flat dashed line correspond to the case $\bar {\mathcal {C}}_{Dip}=0$

We remark that if *γ* is given by the Stokes’ law (15) then the collision time *T*_*d*_ does not depend on the viscosity of the medium surrounding the particles but only on the ratio between the radii of the *A*- and *B*-type particles, on the functional form of the interaction potential between the obstacles, and on the strength of this potential. As in the previous section, we choose identical *A*- and *B*-particles in order to introduce adimensional units. For a potential of the form $U=\mathcal {C} r^{-n}$, Eq. (42) is rewritten as follows:

(53) $$ y(d=Rl) = \frac{1}{1 + \frac{4 d \,F(d)\gamma_{A}}{3 k T\gamma_{B} \left(1 +\sqrt{1+8\,\frac{\gamma_{A}}{\gamma_{B}}}\right)^{2}}} = \frac{1}{1+\frac{1}{12}n\bar{\mathcal{C}}l^{-n}}  $$

as *γ*_*A*_=*γ*_*B*_. In Figs. 5 and 6 we report the different patterns obtained for *D*/*D*_0_ relative to the tracers (*A*-particles) as a function of the average distance *d* between any pair of obstacles (*B*-particles) interacting through the Coulombic and dipolar potential.

## Conclusions

The main aim of this paper is to give an analytical estimation of the self-diffusion coefficient of passive tracers as a function of the concentration and strength of mutual interaction of obstacles. We considered a very simple model of passive tracers and interacting obstacles diffusing in a low concentration limit. The diffusion law was assumed to be Brownian both for the tracers and the obstacles. Nevertheless, it would certainly be interesting in further studies to consider also other diffusive laws, in order to refine the model for crowded systems [20]. The CTRW framework is well suited for this, as it has been discussed in Section “Methods: continuous time random walk formalism”.

We found that the value of the Brownian self-diffusion coefficient of passive tracers is markedly affected by the randomly moving obstacles. The effects related to the presence and the strength of interactions among obstacles is in general less important than the “effective dynamical excluded volume” related to the friction constant. We stress that this result strongly depends on our estimation of the free-flight time *T*_*d*_ of passive tracers, which is quite crude and seems to be the main aspect to be refined in our model in order to obtain more accurate results. An attempt to modify the estimation of *T*_*d*_ is suggested in this article, resulting in the so called rescaled free-flight time distribution; in this case, the effect of friction is neglected and the slowing down of the passive tracers diffusion is due to only to the concentration of the obstacles and the strength of their mutual interactions. Nevertheless, this model has not yet a clear correspondence to real biological models.

Although we have adopted strong approximations and simplifications with respect to a realistic biological case of crowding, this work represents a first step in the analytic study of the value of the diffusion coefficient of passive tracers in the presence of interacting obstacles, and this fact can have relevant prospective consequences for applications to biology. For instance, the description of the complex network of biochemical reactions taking place in living cells could be markedly affected by the activation of long-range intermolecular interactions of the kind discussed in Ref. [15]. In particular, if we imagine a cytoplasm crowded by biomolecules interacting at a long distance, then molecules that would be driven to their targets only by diffusion could be considerably slowed down.

## Appendix

In this section, we compute the probability distribution *P*(**r**,*t*) for the walker to be at location **r**, at time *t*, following [17] and generalising the result to the three-dimensional case.

Let *ψ*(**r**,*t*) be, as in Section “Methods: continuous time random walk formalism”, the probability density of making a displacement **r** in time *t* in a single motional event:

$$\psi(\textbf{r}, t) = \Lambda(\textbf{r})\; \delta\left(t - \frac{\left|\textbf{r}\right|}{v_{0}}\right) $$

The probability *Q*(**r**,*t*) of arriving at location **r** exactly at time *t* and to stop before randomly choosing a new direction satisfies the recursion relation:

$$Q(\textbf{r}, t) = \int_{0}^{+\infty} dt' \int d^{3}\textbf{r'} \;Q(\textbf{r}-\textbf{r'}, t-t')\;\psi(\textbf{r'}, t') + \delta(\textbf{r})\delta(t) $$

### Jump model

In the Jump Model, particles wait at a particular location before moving instantaneously to the next one, the displacement being chosen according to the probability density *Λ*(**r**), the waiting time before the jump being |**r**|/*v*_0_ (because of the *δ*-function in the expression of *ψ*(**r**,*t*)).

The three-dimensional formulation is straightforward in this case (and it appears for example in [18]). We have for the probability distribution *P*(**r**,*t*):

$$P(\textbf{r}, t) = {\int_{0}^{t}} dt' \;Q(\textbf{r}, t-t')\;\Psi(t') $$

where *Ψ*(*t*) is the probability for not leaving a position up to time *t*:

$$\Psi(t) = \int_{t}^{+\infty} dt' \int d^{3}\textbf{r} \;\psi(\textbf{r}, t') $$

Passing to the Fourier-Laplace transform defined by:

$$f(\textbf{k}, s) = \int_{0}^{+\infty} dt\,e^{-st} \int d^{3}\textbf{r}\,e^{i \textbf{k}\cdot\textbf{r}} \,f(\textbf{r},t) $$

we get

$$Q(\textbf{k}, s) = \frac{1}{1 - \psi(\textbf{k},s)} $$

so that

$$P(\textbf{k}, s) = \frac{\Psi(s)}{1 - \psi(\textbf{k},s)} $$

The mean square displacement 〈*r*^2^(*t*)〉 is the inverse Laplace transform of the quantity

(54) $$  {}\langle r^{2}(s)\rangle = -\!\left.\Delta_{\textbf{k}} P(\textbf{k}, s)\right|_{\textbf{k}=0} \!= -\left.\frac{\Psi(s)}{(1 - \psi(\textbf{k},s))^{2}}\left[ \Delta_{\textbf{k}}\psi(\textbf{k},s) +\! \frac{2}{1 - \psi(\textbf{k},s)} (\nabla_{\textbf{k}}\psi(\textbf{k},s))^{2} \right]\right|_{\textbf{k}=0}  $$

where *Δ*_**k**_ is the Laplacian ($\Delta _{\textbf {k}} = \partial ^{2}/\partial {k_{x}^{2}} + \partial ^{2}/\partial {k_{y}^{2}} + \partial ^{2}/\partial {k_{z}^{2}}$) and ∇_**k**_ is the gradient (∇_**k**_=(*∂*/*∂k*_*x*_,*∂*/*∂k*_*y*_,*∂*/*∂k*_*z*_)).

We now use the fact that in our case diffusion is isotropic. As discussed in Section “Methods: continuous time random walk formalism”, this allows to write

$$\psi(\textbf{r},t) = \frac{\phi(t)}{4\pi\,(v_{0} t)^{2}}\,\delta(\left|\textbf{r}\right| - v_{0} t) $$

where we have introduced the waiting time distribution *ϕ*(*t*), which is the probability density function that a single motional event has duration *t*, and is normalised by $\int _{0}^{+\infty } dt\,\phi (t) = 1$. It is easy to show that

(55) $$  \Psi(s) = \frac{1 - \phi(s)}{s}  $$

(56) $$  \psi(\textbf{k}=0,s) = \phi(s)  $$

(57) $$  \Delta_{\textbf{k}}\psi(\textbf{k},s)|_{\textbf{k}=0}= - \, {v_{0}^{2}}\frac{\mathrm{d}^{2}}{\mathrm{d}s^{2}}\phi(s)  $$

where *ϕ*(*s*) is the Laplace transform of *ϕ*(*t*).

Isotropy implies that ∇_**k**_*ψ*(**k**,*s*)|_**k**=0_=0, so that, replacing Eqs. (55), (56), (57) in Eq. (54) we get

(58) $$  \langle r^{2}(s)\rangle = \frac{{v_{0}^{2}}}{(1 - \phi(s))\,s}\cdot\frac{\mathrm{d}^{2}}{\mathrm{d}s^{2}}\phi(s)  $$

Expanding expression (58) around *s*≃0, we obtain

$$\langle r^{2}(s)\rangle \simeq\frac{{v_{0}^{2}} \langle t^{2}\rangle_{\phi}}{s^{2}\langle t \rangle_{\phi}} $$

Using Tauberian theorems [21], which relate the behavior of a function *f*(*t*) at large *t* to that of its Laplace transform at small *s*, we have at large times:

$$\langle r^{2}(t)\rangle \simeq \frac{{v_{0}^{2}}\langle t^{2}\rangle_{\phi}}{\langle t\rangle_{\phi}}\;t $$

which is the same as Eq. (10) of Section “Methods: continuous time random walk formalism”.

### Velocity model

In the Velocity Model, each walker moves with constant velocity *v*_0_ between turning points where a new direction and a new distance of flight are chosen according to the probability density *Λ*(**r**). We have in this case:

$$P(\textbf{r}, t) = {\int_{0}^{t}} \mathrm{d}t' \int\mathrm{d}^{3}\textbf{r'}\;Q(\textbf{r}-\textbf{r}', t-t')\;\Psi(\mathbf{r}',t') $$

where *Ψ*(**r**,*t*) represents the probability for a particle to make a displacement **r** in a time *t* in a single motional event and without stopping at time *t*. The explicit expression for *Ψ*(**r**,*t*) in three dimensions is given by:

$$ \Psi(\textbf{r},t)=p_{\alpha,\beta}(r|t)\int \mathrm{d}^{3} \textbf{r}'\int_{0}^{+\infty}\mathrm{d}t'\;\psi(\textbf{r}',t')\theta(\left|\textbf{r}'\right|-\left|\textbf{r}\right|)\theta(t'-t)\delta(\alpha'-\alpha)\delta(\beta'-\beta) $$

where *α*,*α*^′^,*β*,*β*^′^ are the angles which define the direction of vectors **r** and **r**^′^ in a polar reference system and *p*_*α*,*β*_(*r*|*t*) is the conditional probability of making a displacement of distance *r* in a time interval *t* along a vector whose orientation is specified by the angles *α* and *β*. Heaviside functions *θ*(*x*) take into account time ordering *t*^′^>*t* so that |**r**^′^|−|**r**|>0, as the velocity is constant.

We again consider the Fourier-Laplace transform of the previous functions, obtaining:

$$Q(\textbf{k}, s) = \frac{1}{1 - \psi(\textbf{k},s)} $$

and

$$P(\textbf{k}, s) = \frac{\Psi(\textbf{k},s)}{1 - \psi(\textbf{k},s)} $$

The mean square displacement 〈*r*^2^(*t*)〉 as a function of time is the inverse Laplace transform of the quantity:

(59) $$ {} \begin{aligned}  & \langle r^{2}(s)\rangle = -\Delta_{\mathbf{k}=0}P(\mathbf{k},s)|_{\mathbf{k}=0}=-\left[\frac{\Delta_{\mathbf{k}} \Psi(\mathbf{k},s)}{(1-\psi(\mathbf{k},s))}+ \frac{2\nabla_{\mathbf{k}}\psi(\mathbf{k},s)\cdot\nabla_{\mathbf{k}}\Psi(\mathbf{k},s)}{(1-\psi(\mathbf{k},s))^{2}}\right.\\ &\left.\, \, \, \qquad \qquad \qquad \qquad \qquad \qquad+\frac{2\Psi(\mathbf{k},s)|\nabla_{\mathbf{k}}\psi(\mathbf{k},s)|^{2}}{(1-\psi(\mathbf{k},s))^{3}}+\frac{\Psi(\mathbf{k},s)\Delta_{\mathbf{k}}\psi(\mathbf{k},s)}{(1-\psi(\mathbf{k},s))^{2}} \right]\left.\right|_{\mathbf{k}=0} \end{aligned}  $$

As we consider the isotropic case, we can rewrite *ψ*(**r**,*t*) as

$$\psi(\textbf{r},t) = \frac{\phi(t)}{4\pi\,(v_{0} t)^{2}}\,\delta(\left|\textbf{r}\right| - v_{0} t) $$

Under this hypothesis *Ψ*(**r**,*t*) has the form:

$$  \Psi(\textbf{r},t)=\frac{\delta(|\textbf{r}|-v_{0} t)}{{v_{0}^{2}} t^{2}}\int \mathrm{d}^{3} \textbf{r}'\int_{0}^{+\infty}\mathrm{d}t' \frac{\phi(t')}{4 \pi {v_{0}^{2}} t'^{2}}\delta(|\textbf{r}'| - v_{0} t')\times \theta(\left|\textbf{r}'\right|-\left|\textbf{r}\right|)\theta(t'-t)\delta(\alpha'-\alpha)\delta(\beta'-\beta) $$

The isotropy hypothesis implies ∇_**k**_*ψ*(**k**,*s*)|_**k**=0_=0. Equation (59) then reduces to:

(60) $$ \langle r^{2}(s)\rangle = -\frac{1}{(1-\psi(\textbf{k},s))}\left[\Delta_{\textbf{k}}\Psi(\textbf{k},s) +\frac{\Psi(\textbf{k},s)\Delta_{\textbf{k}}\psi(\textbf{k},s)}{(1-\psi(\textbf{k},s))} \right]\left|\vphantom{\frac{1}{2}}\right._{\textbf{k}=0} $$

It is easy to show that:

(61) $$  \Psi(\textbf{k}=0,s)=\frac{1-\phi(s)}{s}  $$

and

(62) $$  \Delta_{\textbf{k}}\Psi(\textbf{k},s)|_{\textbf{k}=0}=-\,{v_{0}^{2}}\,\frac{\mathrm{d}^{2}}{\mathrm{d}s^{2}}\left[\frac{1-\phi(s)}{s}\right]  $$

where *ϕ*(*s*) is the Laplace transform of *ϕ*(*t*).

Replacing the Fourier-Laplace transforms (56), (57), (61), (62) in Eq. (60) we obtain:

(63) $$ \langle r^{2}(s)\rangle = \frac{{v_{0}^{2}}}{(1-\phi(s))}\left[\frac{\mathrm{d}^{2}}{\mathrm{d}s^{2}}\left(\frac{1-\phi(s)}{s}\right) +\frac{1}{s}\frac{\mathrm{d}^{2}}{\mathrm{d}s^{2}}\phi(s)\right] $$

Expanding expression (63) around zero, we obtain:

$$\langle r^{2}(s)\rangle \simeq\frac{{v_{0}^{2}} \langle t^{2}\rangle_{\phi}}{s^{2}\langle t \rangle_{\phi}} $$

and using Tauberian theorems [21], we have at large times:

$$\langle r^{2}(t)\rangle \simeq\frac{{v_{0}^{2}} \langle t^{2}\rangle_{\phi}}{\langle t \rangle_{\phi}} \, t $$

as in the Jump Model.
